# Role of mitochondrial fusion proteins MFN2 and OPA1 on lung cellular senescence in chronic obstructive pulmonary disease

**DOI:** 10.1186/s12931-023-02634-9

**Published:** 2023-12-18

**Authors:** Chenfei Li, Qi Liu, Qing Chang, Meiqin Xie, Jiali Weng, Xiaohui Wang, Mengnan Li, Jiani Chen, Yan Huang, Xiaohua Yang, Kai Wang, Na Zhang, Kian Fan Chung, Ian M. Adcock, Hai Zhang, Feng Li

**Affiliations:** 1 Department of Pulmonary and Critical Care Medicine, Shanghai Chest Hospital, Shanghai Jiao Tong University School of Medicine, NO.241, West HuaiHai Road, 200030 Shanghai, People’s Republic of China; 2 College of Public Health, University of South China, NO.28, West Changsheng Road, Hengyang, 421001 Hunan People’s Republic of China; 3 School of Pharmacy, Anhui Medical University, Meishan Road, Hefei, 230032 Anhui People’s Republic of China; 4 Department of Central Laboratory, Shanghai Chest Hospital, Shanghai Jiao Tong University School of Medicine, NO.241, West HuaiHai Road, Shanghai, 200030 People’s Republic of China; 5 Airway Disease Section, National Heart and Lung Institute, Imperial College, Dovehouse Street, London, SW3 6LY UK

**Keywords:** Chronic obstructive pulmonary disease (COPD), Mitochondrial dynamics, Mitophagy, Lung senescence, Cigarette smoke

## Abstract

**Background:**

Mitochondrial dysfunction and lung cellular senescence are significant features involved in the pathogenesis of chronic obstructive pulmonary disease (COPD). Cigarette smoke (CS) stands as the primary contributing factor to COPD. This study examined mitochondrial dynamics, mitophagy and lung cellular senescence in COPD patients and investigated the effects of modulation of mitochondrial fusion [mitofusin2 (MFN2) and Optic atrophy 1 (OPA1)] on CS extract (CSE)-induced lung cellular senescence.

**Methods:**

Senescence-associated secretory phenotype (SASP) component mRNAs (IL-1β, IL-6, CXCL1 and CXCL8), mitochondrial morphology, mitophagy and mitochondria-related proteins (including phosphorylated-DRP1(p-DRP1), DRP1, MFF, MNF2, OPA1, PINK1, PARK2, SQSTM1/p62 and LC3b) and senescence-related proteins (including P16, H2A.X and Klotho) were measured in lung tissues or primary alveolar type II (ATII) cells of non-smokers, smokers and COPD patients. Alveolar epithelial (A549) cells were exposed to CSE with either pharmacologic inducer (leflunomide and BGP15) or genetic induction of MFN2 and OPA1 respectively.

**Results:**

There were increases in mitochondrial number, and decreases in mitochondrial size and activity in lung tissues from COPD patients. SASP-related mRNAs, DRP1 phosphorylation, DRP1, MFF, PARK2, SQSTM1/p62, LC3B II/LC3B I, P16 and H2A.X protein levels were increased, while MFN2, OPA1, PINK1 and Klotho protein levels were decreased in lung tissues from COPD patients. Some similar results were identified in primary ATII cells of COPD patients. CSE induced increases in oxidative stress, SASP-related mRNAs, mitochondrial damage and dysfunction, mitophagy and cellular senescence in A549 cells, which were ameliorated by both pharmacological inducers and genetic overexpression of MFN2 and OPA1.

**Conclusions:**

Impaired mitochondrial fusion, enhanced mitophagy and lung cellular senescence are observed in the lung of COPD patients. Up-regulation of MFN2 and OPA1 attenuates oxidative stress, mitophagy and lung cellular senescence, offering potential innovative therapeutic targets for COPD therapy.

**Graphical Abstract:**

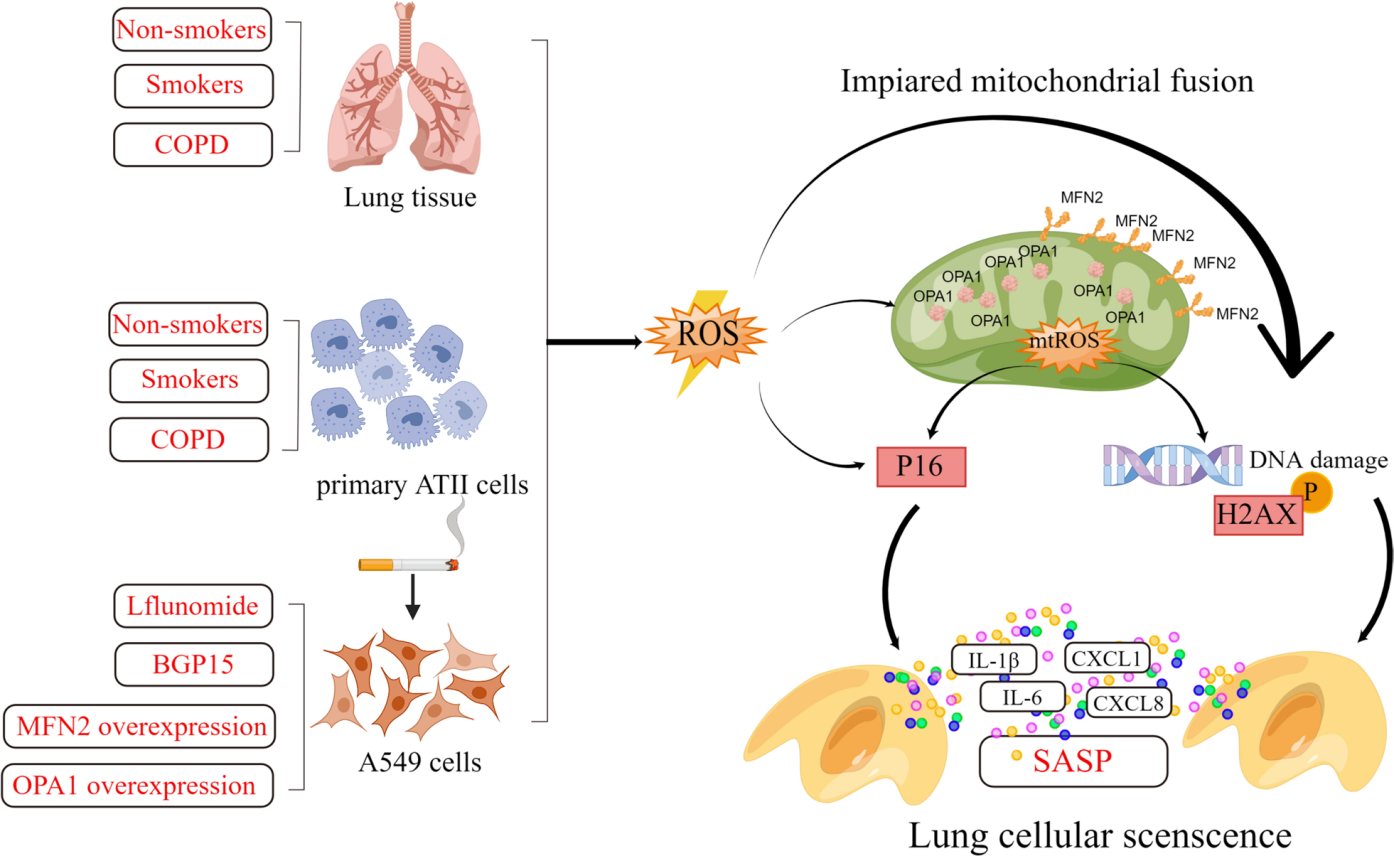

**Supplementary Information:**

The online version contains supplementary material available at 10.1186/s12931-023-02634-9.

## Introduction

Chronic obstructive pulmonary disease (COPD) is marked by a progressive decline in lung function coupled with irreversible airflow obstruction. This condition is a growing worldwide health challenge and ranks as the third most prevalent cause of mortality on a global scale [1]. Cigarette smoke (CS) stands as the primary contributing factor to COPD. As most COPD patients are elderly, and the anatomical and physiological features of lung aging exist in COPD patients, it is reasonable to refer to COPD as an aging-related disease [2]. Both the impairment of mitochondrial function and the premature aging of the lungs have been suggested as pivotal factors in the development of COPD [3, 4].

Mitochondrial dynamics and mitophagy are two main mechanisms by which mitochondrial quality and homeostasis are maintained [3]. Two key proteins namely mitofusin 1 (MFN1) and mitofusin 2 (MFN2) orchestrates the fusion of the outer mitochondrial membranes, while optic atrophy 1 (OPA1) takes charge of merging the inner mitochondrial membranes [5]. Conversely, the interaction between dynamin-related protein 1 (DRP1) and four receptor proteins orchestrates mitochondrial fission [5]. The PTEN-induced kinase 1 (PINK1)–Parkinson Disease protein 2 (PARK2 or PRKN) pathway is a well-established mechanism for mitophagy [6]. During mitophagy, damaged mitochondria are targeted for degradation. PARK2 is recruited and ubiquitinates specific proteins on the mitochondrial surface. This occurrence triggers the mobilization of protein sequestosome-1 (SQSTM1/p62) towards the mitochondria, facilitating a direct engagement with microtubule-associated protein 1A/1B-light chain 3 (LC3), thereby instigating the commencement of autophagosome formation [6].

CS exposure induces mitochondrial dysfunction and triggers mitophagy, promoting mitochondrial injury and airway epithelial cell injury [7]. In our earlier investigation, it was revealed that CS extract (CSE) has the ability to enhance DRP1 and MFF and to reduce the expressions of MFN2 and OPA1 within lung alveolar type II (ATII) cells (A549) [8]. The crucial function of mitochondrial fusion in maintaining mitochondrial quality control has been confirmed by the employment of pharmaceutical compounds that promote mitochondrial fusion, like leflunomide and BGP-15 [9, 10], but their role in COPD mitochondrial dynamics remains unknown.

Accumulation of senescent cells may contribute to aging-related diseases. Cellular senescence is mainly regulated by P53- and P16-mediated pathways [11]. In the P53-mediated pathway, DNA damage or telomere shortening activates DNA repair kinase ataxia-telangiectasia mutated proteins (ATM), which phosphorylates H2A histone family member X (H2A.X, a histone H2A variant), and then activates P53 and P21, leading to cellular senescence. In the P16-mediated pathway, oxidative stress activates P16 which phosphorylates the retinoblastoma (Rb) protein, leading to the activation of P21 and cellular senescence. Ultimately, both routes lead to the buildup of senescent cells and the release of cytokines and chemokines recognized as the senescence-associated secretory phenotype (SASP) response [12]. Components of SASP include interleukin (IL)-1, IL-6, chemokine (C-X-C motif) ligand 1 (CXCL)1 and CXCL8, which are all increased in COPD [12]. Mice exposed to CS showed an increased expression of P16 in the lung [13]. Klotho may function as an anti-aging protein in aging-related diseases guarding against inflammation and oxidative stress [14], and its presence is diminished in airway epithelial cells of individuals with COPD [15].

We hypothesized that exposing to CS could induce mitochondrial dysfunction by reducing MFN2 and OPA1 proteins leading to lung cellular senescence and that up-regulation of mitochondrial fusion proteins might prevent lung cellular senescence. In order to validate this hypothesis, we conducted a targeted investigation into the regulatory impacts of MFN2 and OPA1 on SASP and biomarkers of cellular senescence in CSE-exposed A549 cells by pharmacological induction and genetic overexpression (OE).

## Materials and methods

A detailed ‘materials and methods’ is in the online supplement (see Additional file 1).

### Collection of human lung tissues and culture of primary ATII cells

This research was approved by the Institutional Ethics Committee (No. KS1969) and written informed consent was signed by each subject in accordance with the Declaration of Helsinki. Lung tissues were obtained from newly diagnosed COPD patients or control subjects of no history of obstructive airways diseases with lung nodules or suspected lung cancer undergoing lung resection between July and August 2020 in Shanghai Chest hospital following a previous protocol [16]. A total of 40 subjects were divided into non-smokers (n = 10), smokers without obstruction (n = 10) and COPD (n = 20) patients through a questionnaire and lung function tests. As the COPD patients and smokers were mostly male, we made all subjects including controls men in our research to avoid the influence by gender. Both smokers and COPD patients were active smokers.

Isolation of primary ATII cells was carried out using resected lung tissues in sterile condition. In brief, the lung pieces were minced with scissors and incubated in a solution containing trypsin (Gibco) and collagenase type I (Life technologies) for digestion, which was stopped using DNase I (KeyGen biotechnology), and then filtered through cell strainers at the size of 150 μm and 75 μm in tandem to collect the crude cell suspension. The residual lung tissues were digested and filtered again. The cell suspensions obtained from the two filtrations were centrifuged and resuspended with DMEM/F12 complete medium, and incubated at 37 °C, 5% CO_2_ for 1–2 h. The unadhered cells were aspirated, which was repeated three times, and then the ATII cells were gently collected and added to the culture dish coated with mouse IgG (Sigma) for 3 h, the unadhered cells were aspirated. Cells were resuspended in DMEM/F12K medium with 20% fetal bovine serum (FBS), 200U/ml penicillin and 200 µg/ml streptomycin. The medium was changed every other day. The cells were cultured until they were in good condition, and then subsequent experiments were performed.

### Transmission electron microscopy (TEM) analysis in ATII cells of lung tissues

Lung tissue fragments were initially preserved using 2.5% glutaraldehyde, and subsequently exposed to 1% osmium tetroxide. Following dehydration, the tissue specimens were soaked and embedded in a solution composed of propylene oxide and SPI-pon812 embedding agent (SPI supplies, West Chester, PA, USA). Following high-temperature polymerization, ultrathin sections, ranging from 70 to 80 nanometers in thickness, were treated with uranyl acetate and lead citrate staining before being scrutinized using TEM (JEOL-1400 flash, Akishima, Tokyo, Japan). The evaluation of mitochondrial morphology and the number of authophagosome in ATII cells were conducted utilizing Image J software (National Institute of Health, Bethesda, USA). Freehand tool was used to trace the outer mitochondrial membrane of each mitochondrion to measure area, circularity and perimeter while a straight line along the major axis of each mitochondrion was drawn to measure length.

### Cell line culture, CSE preparation and exposure, pharmacologic and genetic induction

The culture of A549 cells (Shanghai Institutes for Biological Sciences, China Academy of Science, Shanghai) and freshly prepared CSE followed the methods outlined in a prior description [8]. Based on a preliminary study, A549 cells were subjected to 10% concentration of CSE to initiate cellular damage. Prior to this, cells were pretreated with either 10 µM of leflunomide (MFN2 promoter) (#S1247, Selleck, Shanghai, China) or 15µM BGP15 (OPA1 promoter) (#S8370, Selleck) for a duration of 2 h. Following this pre-treatment, the cells were subsequently exposed to vehicle or CSE for another 24 h. Lentivirus transduction was used for genetic induction. The plasmid sequences for human MFN2 overexpression (MFN2 OE) and OPA1 overexpression (OPA1 OE) were obtained from Lncbio-technology (Xuhui, Shanghai, China).

### Cell viability and cell proliferation assay

Cell Counting Kit-8 (CCK8, Dojindo, Kumamoto, Japan) was conducted to assess cell viability, while EdU Cell Proliferation Kit with DAB (Beyotime, Shanghai, China) was utilized to evaluate cell proliferation. Both analyses were conducted in accordance to the manufacturer’s instructions, comparing responses to either vehicle or CSE.

### Measurement of intracellular ROS and mitochondrial ROS (mtROS) and in cells

DCFH-DA (Sigma-Aldrich, St. Louis, MO, USA) was performed to examine the level of intracellular ROS and Mito SOX Red (Invitrogen, Life Technologies, Carlsbad, CA, USA) was employed for mitochondrial ROS (mtROS) respectively as previously described [8].

### Quantitative real-time PCR

TRIzol (Vazyme, Nanjing, Jiangsu, China) was used for isolation of total RNA from both human lung tissues and A549 cells. Subsequently, the RNA’s concentration and purity were evaluated. ChamQ Universal SYBR qPCR Master Mix (Vazyme, Nanjing, Jiangsu, China) was employed for quantitative real-time PCR for SASP components, utilizing an ABI ViiATM 7 System. The primer sequences for the cytokines as well as β-actin were documented in Table 1.

**Table 1:** Primer sequences of cytokines and β-actin

IL-1β	Forward	5′-TCGCAGCAGCACATCAACAAGAG-3′
	Reverse	5′-AGGTCCACGGGAAAGACACAGG-3′
IL-6	Forward	5′-CACTGGTCTTTTGGAGTTTGAG-3′
	Reverse	5′-GGACTTTTGTACTCATCTGCAC-3′
CXCL1	Forward	5′-AAGAACATCCAAAGTGTGAACG-3
	Reverse	5′-CACTGTTCAGCATCTTTTCGAT-3′
CXCL8	Forward	5′-AACTGAGAGTGATTGAGAGTGG-3′
	Reverse	5′-ATGAATTCTCAGCCCTCTTCAA-3′
β-actin	Forward	5′-GGCCAACCGCGAGAAGATGAC-3′
	Reverse	5′-GGATAGCACAGCCTGGATAGCAAC-3′

### Western Blot analysis in lung tissues and cells

By homogenization and lysis in RIPA Lysis Buffer (Beyotime), we extracted total proteins from lung tissues, primary ATII cells, and A549 cells. The protein content was quantified using a BCA kit (Beyotime). Western Blot analysis in lung tissues and cells was performed against DRP1, phosphorylated-DRP1 (p-DRP1) (Ser616), MFF, OPA1, MFN2 (1:1000, Cell Signaling Technology, Danvers, MA, USA), PINK1, PARK2, SQSTM1/p62, LC3b, P16, H2AX (1:1000, Abcam Cambridge, MA, USA), Klotho and GAPDH (1:1000, Proteintech, Wuhan, Hubei, China). Bands were developed by ECL chemiluminescent substrate (Millipore, Billerica, MA, USA).

### Mitochondrial potential, mitophagy activity and morphology.

The cells were stained using JC-1 (Thermo Fisher Scientific, MA, USA) for membrane potential, mitophagy detection kit (Dojindo, Kumamoto, Japan) for mitophagy activity, and MitoTracker Green (Beyotime, Shanghai, China) and 4′,6-diamidino-2-phenylindole (DAPI, Beyotime) for morphology following the instructions provided by the manufacturer. The mitochondrial membrane potential was quantified based on the red/green fluorescence ratio. The mitophagy activity fluorescence and morphology was measured and imaged under a confocal laser microscope (Zeiss, Oberkochen, Germany). The ratio of MitoTracker area to cell area, mitochondrial fragmentation percentage and perinuclear mitochondrial compaction percentage were calculated as previously described [7].

### Mitochondrial respiratory chain (MRC) complexes activities and oxygen consumption rate (OCR)

MRC complexes I, III, and V activities within lung tissues were evaluated using an activity assay kit (Solarbio Life Sciences, Beijing, China) following the provided instructions. The mitochondrial OCR in cells was measured using the standard protocol established for XFe96 Extracellular Flux Analyzer (Seahorse Bioscience, North Billerica, MA, USA) following the manufacturer’s instruction.

### Statistical analysis

Data are presented as mean ± SD. For correlation analysis, Pearson’s test was applied to normally distributed data, while Spearman’s rank test was used for non-normally distributed data. Additionally, Fisher’s exact test was employed for multiple composition ratio comparisons using SPSS software 20.0 (IBM, NY, USA). Utilizing GraphPad Prism 8, a comparison among multiple groups was conducted through One way ANOVA with Bonferroni’ s post hoc test (for equal variance) or Dunnett’ s T3 post hoc test (for unequal variance). Meanwhile, we applied a correction that controlled the false discovery rate using the two-stage step-up method of Benjamini, Krieger, and Yekutieli. p < 0.05 was set as a level considered statistically significant.

## Results

### Clinical features

As shown in Table 2, all of the subjects were male, and there was no noteworthy distinction between groups in relation to age, body mass index (BMI) and pathology, while COPD patients and smokers showed declined lung function and airflow obstruction in comparison to non-smokers.

**Table 2:** Subjects’ clinical characteristics (mean ± SD)

	Non-smokers	Smokers	COPD	F or χ^2^	p value
Female/male	0/10	0/10	0/20	–	–
Age (year),	62 ± 4.7	65 ± 5.6	65 ± 5.8	0.870	0.427
Smoking index (pack-years)	0	49.5 ± 18.3^***^	39.1 ± 23.5^***^	1.488^b^	0.233
Body mass index (BMI, kg/m^2^)	23.9 ± 3.6	21.6 ± 2.9	22.8 ± 2.7	1.245	0.300
FVC (%pred)	94.3 ± 17.6	90.0 ± 13.1	86.25 ± 11.8	1.169	0.322
FEV_1_ (%pred)	101.3 ± 12.2	92.0 ± 12.1	72.8 ± 11.9^***##^	21.163	< 0.001
FEV_1_/FVC	84.3 ± 6.0	77.0 ± 9.8	63.2 ± 6.7^***###^	30.323	< 0.001
FRC (%pred)	93.3 ± 21.6	113.1 ± 17.1	128.2 ± 27.0^**^	6.878	0.003
TLC (%pred)	113.3 ± 11.4	124.3 ± 12.6	129.0 ± 12.4^**^	5.123	0.011
DL_CO_ (%pred)	115.5 ± 16.5	81.9 ± 11.8^**^	73.2 ± 24.6^***^	14.715	< 0.001
Pathology, Non-malignant/ Lung cancer	2/8	2/8	1/19	2.414^a^	0.264
CAT score, mild /moderate /severe	–	10/0/0	7/12/1^##^	12.101^ab^	0.001
mMRC classification, 0/1/2	–	6/4/0	9/10/1	0.969^ab^	0.800

*FVC%pred* forced vital capacity percent predicted, *FEV*_1_*%pred *forced expiratory volume in one second percent predicted, *FRC%pred *functional residual volume percent predicted, *TLC%pred *total lung capacity percent predicted, *DL*_CO_*%pred *diffusing capacity of lung for carbon monoxide percent predicted, *CAT *COPD assessment test, *mMRC *modified Medical Research Council dyspnea score, *VA *alveolar volume

^a^Fisher exact test

^b^COPD vs. Smokers

^**^p < 0.01

^***^p < 0.001 compared to non-smokers

^##^p < 0.01

^###^p < 0.001 compared to smokers

### Mitochondrial morphology and MRC complex activity in lung tissues

**Fig. 1:**
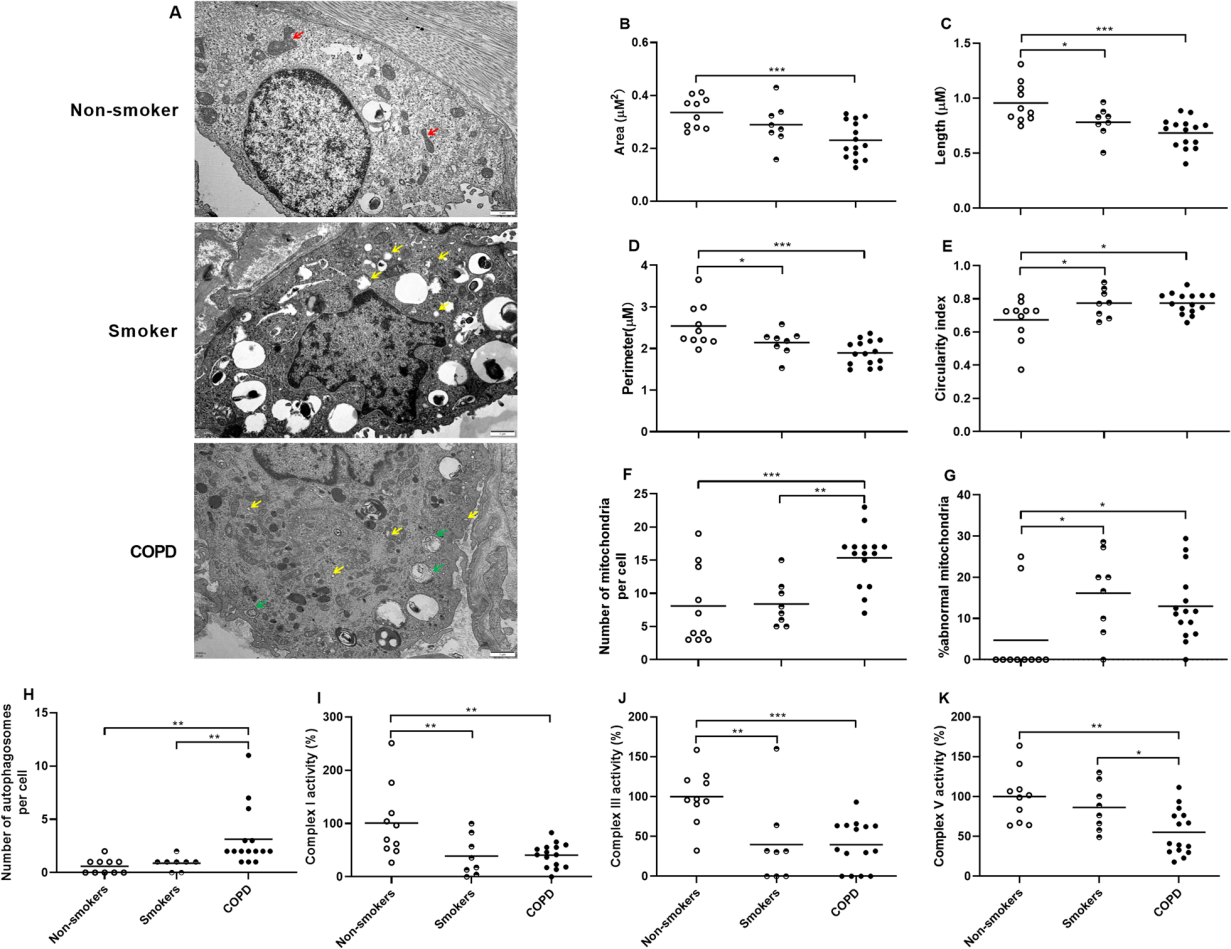
Morphology and function of mitochondria of non-smokers (n = 10), smokers (n = 8) and COPD patients (n = 15). Representative ultrastructure of mitochondrial morphology in the alveolar type II (ATII) cell of non-smoker, smoker and COPD patient is obtained by Transmission electron microscopy (TEM) (**A**). Red arrows indicate elongated mitochondria, yellow arrows indicate abnormal mitochondria (mitochondria with impaired membrane and cristae arrangements) and green arrows indicate autophagosomes. The scale bar is 1 μm. Quantification of mitochondrial morphology including area (**B**), length (**C**), perimeter (**D**), circularity index (**E**), number of mitochondria per cell (**F**), percentage of abnormal mitochondria (**G**) and number of autophagosomes (**H**) is conducted by Image J analysis. The mitochondrial respiratory chain (MRC) complexes I (**I**), III (**J**) and V (**K**) activities are assessed using an activity assay kit, and data are presented as the percentage changes compared to non-smokers. Data are presented as individual and mean values. ^*^p < 0.05, ^**^p < 0.01, ^***^p < 0.001

The representative images of mitochondria and autophagosomes in the ATII cells of non-smoker, smoker and COPD are presented in Fig. 1A. In contrast to non-smokers, COPD patients’ ATII cells exhibited reduced area, length and perimeter of mitochondria (p < 0.001, p < 0.001 and p < 0.001 respectively, Fig. 1B–D) indicative of the shrunken mitochondria. Furthermore, the circularity index of mitochondria was elevated in COPD patients in comparison to non-smokers (p < 0.05, Fig. 1E). In addition, COPD patients showed increased number of mitochondria per cell compared to non-smokers and smokers (p < 0.001 and p < 0.01 respectively, Fig. 1F). Mitochondria in ATII cells of COPD patients demonstrated impaired membranes and cristae arrangements, which were defined as abnormal mitochondria compared to non-smokers. Smokers and COPD patients had higher percentage of abnormal mitochondria and greater numbers of autophagosomes compared to non-smokers (p < 0.05, p < 0.05, p<0.01 and p<0.01, respectively, Fig. 1G-H). COPD patients had reductions in the activities of MRC complexes I, III, and V as opposed to non-smokers (p < 0.01, p < 0.001 and p < 0.01 respectively, Fig. 1I-K). Concurrently, smokers had decreased activities of MRC complexes I and III compared to non-smokers (both p < 0.01, Fig. 1I, J).

### SASP mRNA levels in lung tissues

**Fig. 2:**
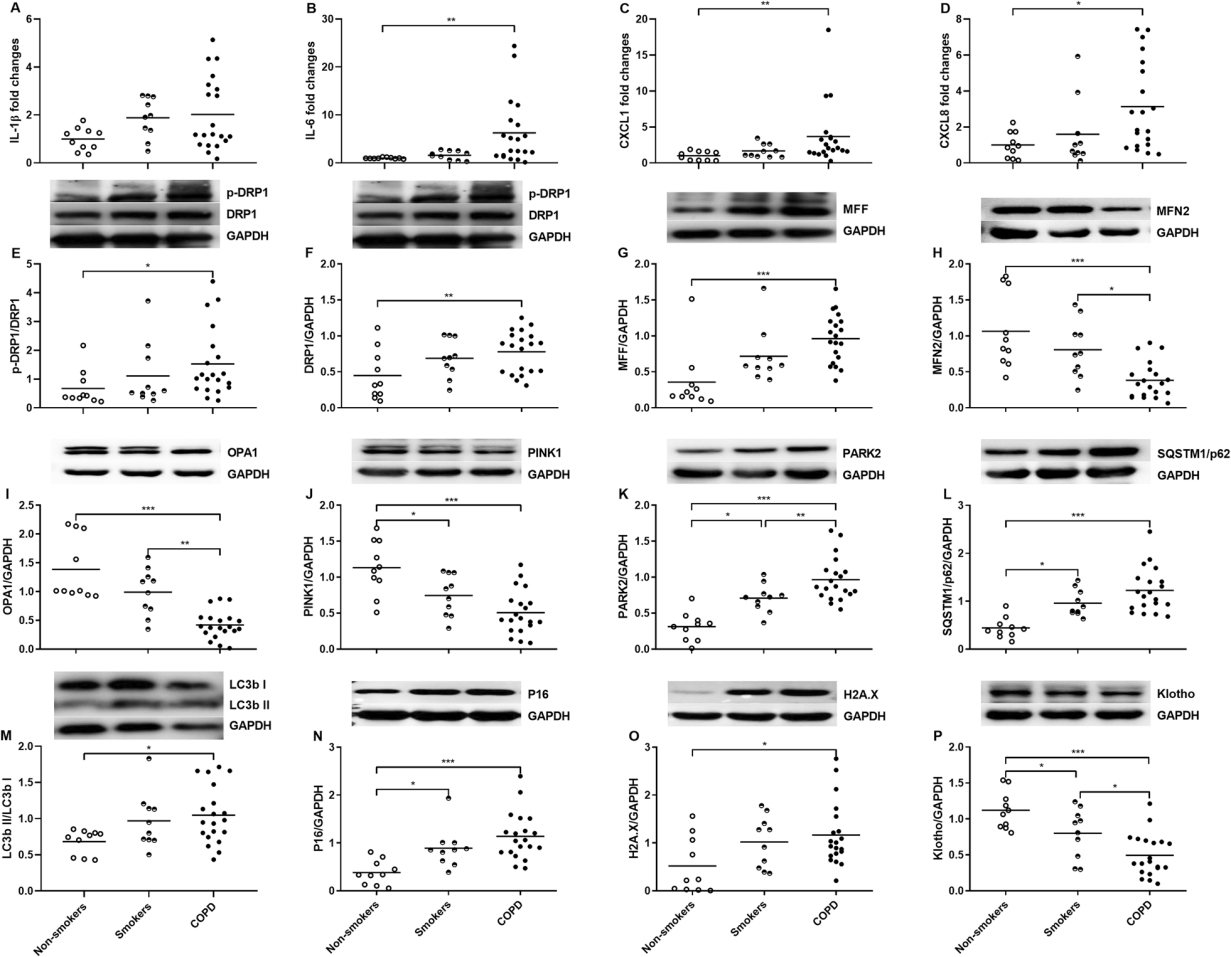
Expression of senescence-associated secretory phenotype (SASP) components mRNA and mitochondria- and senescence-related proteins in human lung tissues of non-smokers (n = 10), smokers (n = 10) and COPD (n = 20) patients. Quantitative real-time PCR is employed to measure the mRNA levels of interleukin (IL)-1β (**A**), IL-6 (**B**), CXCL1 (**C**), and CXCL8 (**D**) in relation to β-actin. Data are displayed as individual and mean values of fold changes compared to non-smokers. Mitochondria-related proteins including phosphorylated-DRP1 (p-DRP1)/DRP1 (**E**), DRP1(**F**), MFF (**G**), MFN2 (**H**), OPA1 (**I**), PINK1 (**J**), PARK2 (**K**) and SQSTM1/p62 (**L**) and LC3b II/ LC3b I (**M**), and senescence-related proteins including P16 (**N**), H2A.X (**O**) and Klotho (**P**) are measured by Western blot. Every panel incorporates an illustrative Western blot image, and data are presented as individual and mean values of each protein. ^*^p < 0.05, ^**^p < 0.01, ^***^p < 0.001

The mRNA expression of IL-1β exhibited an ascending pattern in COPD patients compared to non-smokers (Fig. 2A), whereas IL-6, CXCL1 and CXCL8 mRNA levels were elevated in COPD patients compared to non-smokers (p < 0.01, p < 0.01 and p < 0.05 respectively, Fig. 2B–D). Furthermore, there was no significant difference in SASP mRNA levels between smokers and non-smokers (Fig. 2A–D).

### Mitochondria- and senescence-related proteins in lung tissues

The phosphorylation levels of DRP1 and the protein levels of DRP1 and MFF exhibited an elevation while the protein levels of MFN2 and OPA1 showed a reduction in COPD patients compared to non-smokers (p < 0.05, p < 0.01, p < 0.001, p < 0.001 and p < 0.001 respectively, Fig. 2E–I). The protein levels of PINK1 were decreased, while that of PARK2 and SQSTM1/p62 and ratios of LC3b II/I were increased in both smokers and COPD patients compared to non-smokers, which may indicate an enhanced mitophagy (p < 0.05, p < 0.001, p < 0.05, p < 0.001, p < 0.05, p < 0.001 and p < 0.05 respectively, Fig. 2J–M). COPD patients exhibited heightened protein levels of P16 and H2A.X, along with a diminished Klotho protein level in contrast to non-smokers (p < 0.001, p < 0.05 and p < 0.001 respectively, Fig. 2N–P). Additionally, smokers had elevated P16 protein and reduced Klotho protein compared to non-smokers (p < 0.05 and p < 0.05 respectively, Fig. 2N, P).

### Mitochondrial morphology, mitochondria- and senescence-related proteins in primary ATII cells

**Fig. 3:**
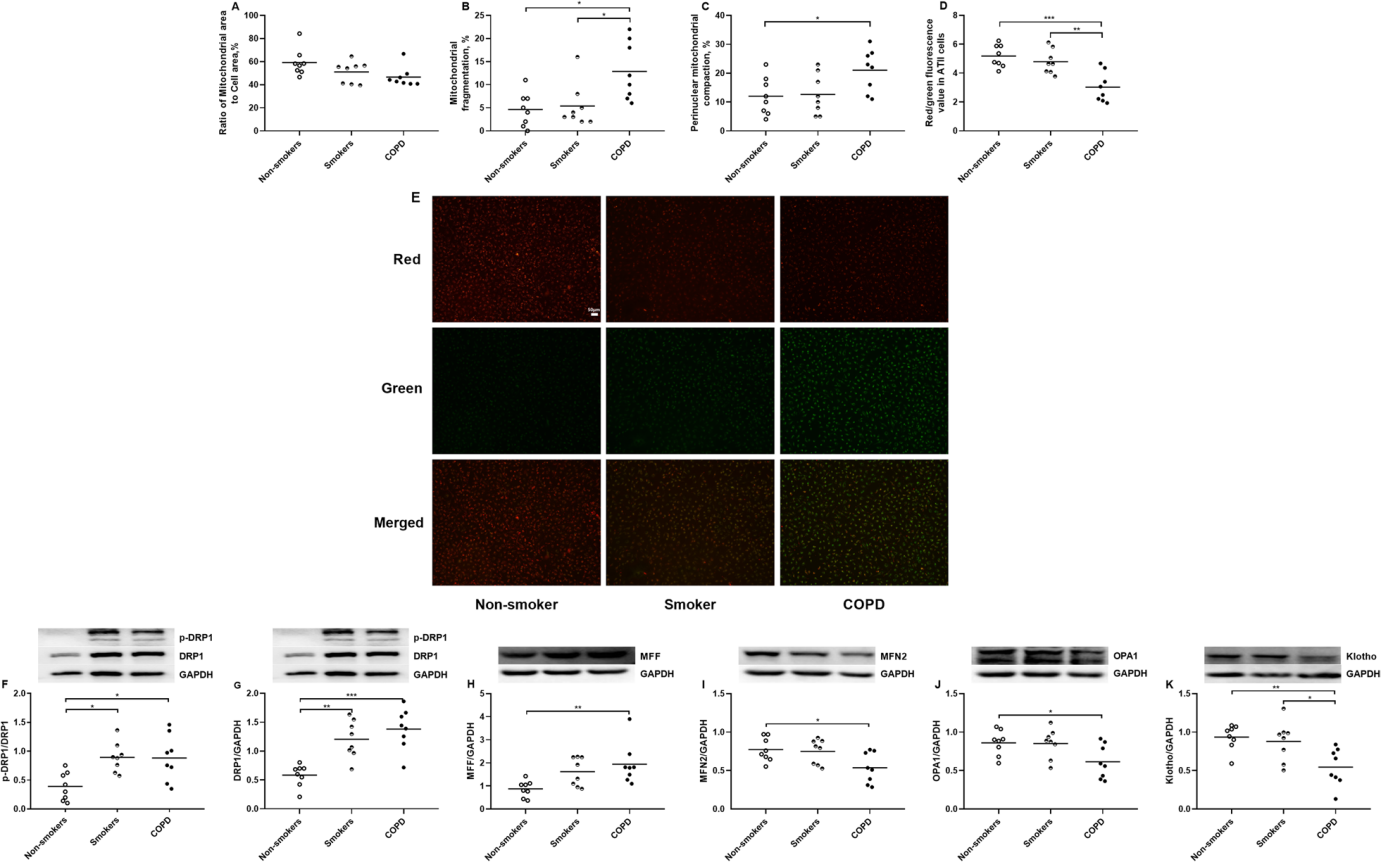
Mitochondrial morphology and mitochondria- and senescence-related proteins expression in primary alveolar type II (ATII) cells of non-smokers, smokers and COPD patients. The area of mitochondria (**A**), and percentage of mitochondrial fragmentation (**B**) and perinuclear mitochondrial compaction (**C**) are assessed by MitoTracker Green. Data are presented as the percentage changes compared to non-smokers. The red/green fluorescence value (**D**) is used to quantify the mitochondrial membrane potential detected by JC-1 staining. N = 8 in each group. Data are presented as individual and mean values. Representative fluorescent microscopy of JC-1 staining in primary ATII cells of non-smoker, smoker and COPD patient (**E**). The scale bar is 50 μm. Mitochondria-related proteins including phosphorylated-DRP1 (p-DRP1)/DRP1 (**F**), DRP1 (**G**), MFF (**H**), MFN2 (**I**), OPA1 (**J**) and senescence-related protein Klotho (**K**) are measured by Western blot. N = 8 in each group. Every panel incorporates an illustrative Western blot image, and data are presented as individual and mean values of each protein. ^*^p < 0.05, ^**^p < 0.01, ^***^p < 0.001

There was no significant difference in the mitochondrial area of primary ATII cells among non-smokers, smokers and COPD patients (Fig. 3A). There was an augmentation in mitochondrial fragmentation in primary ATII cells derived from both smokers and COPD patients compared to non-smokers (p < 0.05 and p < 0.05 respectively, Fig. 3B). The perinuclear mitochondrial compaction was increased in primary ATII cells of COPD patients compared to non-smokers (p < 0.05, Fig. 3C). The membrane potential values were reduced in primary ATII cells of COPD patients compared to smokers and non-smokers (p < 0.001 and p < 0.01 respectively, Fig. 3D). The representative images of membrane potential in primary ATII cells of non-smoker, smoker and COPD patient are shown in Fig. 3E.

The protein ratios of p-DRP1/DRP1 and protein levels of DRP1 and MFF were increased in primary ATII cells from COPD patients compared to non-smokers (p < 0.05, p < 0.001 and p < 0.01 respectively, Fig. 3F**–**H), whilst the protein levels of MFN2 and OPA1 were reduced in COPD patients compared to non-smokers (p < 0.05 and p < 0.05 respectively, Fig. 3I, J). In addition, the primary ATII cells had increased protein ratios of p-DRP1/DRP1 and protein levels of DRP1 in smokers compared to non-smokers (p < 0.05 and p < 0.01 respectively, Fig. 3F, G). The primary ATII cells also had reduced protein levels of Klotho in COPD patients compared to smokers and non-smokers (p < 0.01 and p < 0.05 respectively, Fig. 3K).

### Effects of leflunomide and BGP15 in CSE-exposed A549 cells

**Fig. 4:**
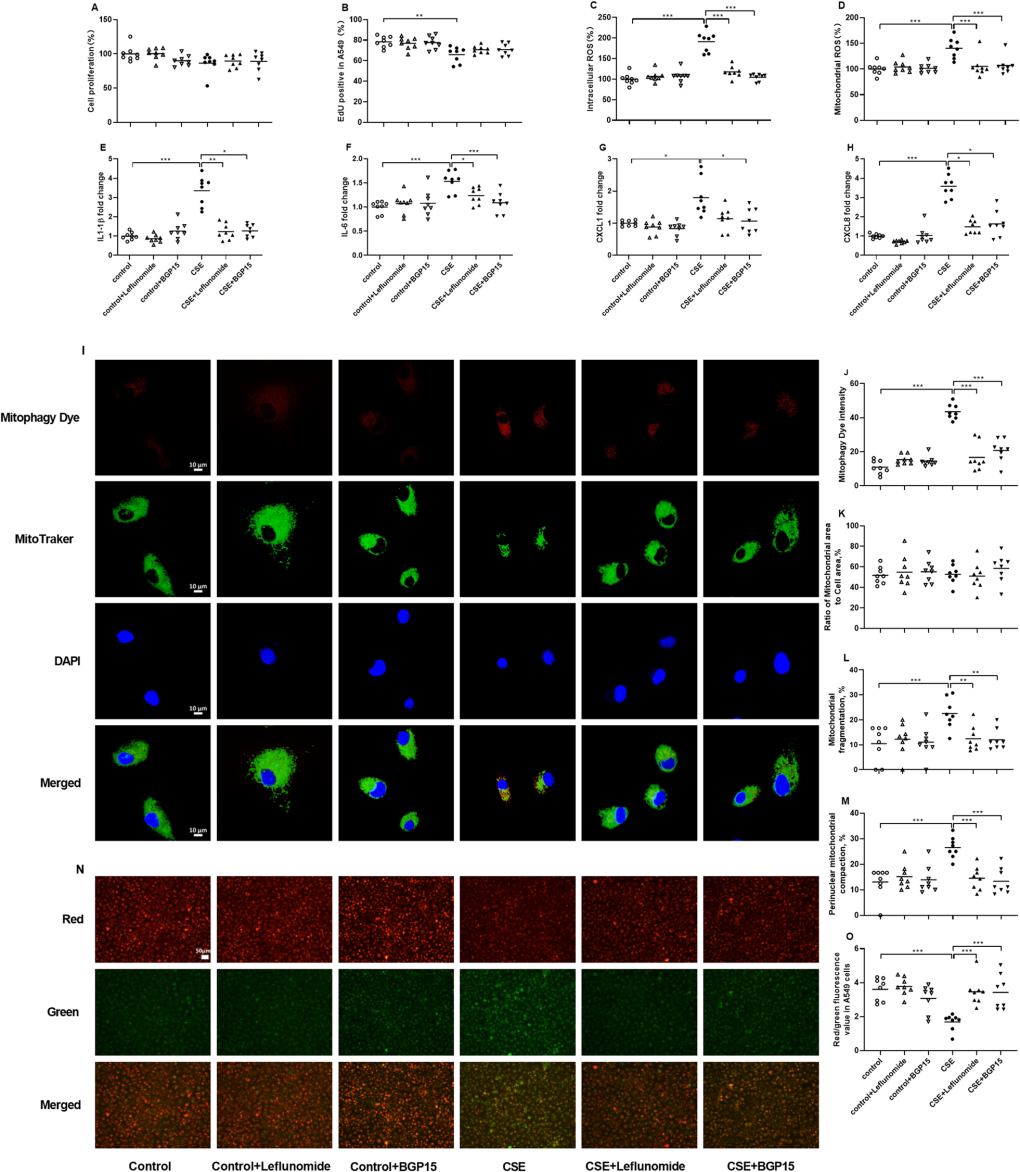
Effects of leflunomide (10µM) and BGP15 (15µM) on cell proliferation, oxidative stress, senescence-associated secretory phenotype (SASP) components mRNA levels and mitochondrial morphology in A549 cells in the presence and absence of cigarette smoke extract (CSE). Cell viability (**A**), cell proliferation (B), intracellular ROS (**C**) and mitochondrial ROS (**D**) are respectively detected by CCK-8, Edu staining, DCFH-DA and Mito SOX Red, and data are presented as percentage changes compared to control cells. Quantitative real-time PCR is employed to measure the mRNA levels of IL-1β (**E**), IL-6 (**F**), CXCL1 **(G)** and CXCL8 (**H**) in relation to β-actin, and data are presented as individual and mean values of fold changes compared to control cells. N = 8 in each group. Representative confocal microscopy images of the mitophagy dye fluorescence and Mito-Tracker Green staining (**I**, original magnification, ×63). The scale bar is 10 μm. The fluorescent intensity of mitophagy (**J**), the area of mitochondria (**K**), and percentage of mitochondrial fragmentation (**L**) and perinuclear mitochondrial compaction (**M**) are assessed by MitoTracker Green. Representative fluorescent microscopy images of JC-1 staining (**N**) and JC-1 values (**O**). The scale bar is 50 μm. N = 8 in each group. Data are presented as individual and mean values. ^*^p < 0.05, ^**^p < 0.01, ^***^p < 0.001

Leflunomide (10µM) and BGP15 (15µM) were used to up-regulate the expression of MFN2 and OPA1 respectively in CSE-exposed A549 cells. No significant difference in the cell viability by CCK8 assay was observed among control cells, leflunomide or BGP15-treated control cells, CSE-exposed cells and leflunomide or BGP15-treated CSE-exposed cells **(**Fig. 4A). The EdU staining assay showed that CSE inhibited the proliferation of A549 cells (p < 0.01) (Fig. 4B). The intracellular ROS and mtROS levels were increased in CSE-exposed cells in comparison to control cells (both p < 0.001, Fig. 4C, D). However, both leflunomide and BGP15 effectively attenuated the CSE-induced levels of intracellular ROS and mtROS (all p < 0.001, Fig. 4C, D).

The mRNA levels of SASP components, encompassing IL-1β, IL-6, CXCL1 and CXCL8, exhibited elevation in CSE-exposed cells (p < 0.001, p < 0.001, p < 0.05 and p < 0.001 respectively, Fig. 4E–H). Both leflunomide and BGP15 effectively restrained the CSE-induced mRNA levels of IL-1β, IL-6 and CXCL8 (p < 0.01, p < 0.05, p < 0.05, p < 0.001, p < 0.05 and p < 0.05 respectively, Fig. 4E, F, H). Notably, only BGP15 exhibited a reduction in the CSE-induced mRNA level of CXCL1 (p < 0.05, Fig. 4G).

The representative images of mitophagy dye and mitochondrial morphology are shown in Fig. 4I. The intensity of mitophagy fluorescence was increased in CSE-exposed cells, and it was reduced by treatment of both leflunomide and BGP-15 (all p < 0.001, Fig. 4J). Mitochondrial area showed no significant difference among different groups of A549 cells (Fig. 4K). Mitochondrial fragmentation and perinuclear mitochondrial compaction were increased in CSE-exposed cells, which were inhibited by treatment with both leflunomide and BGP-15 (p < 0.001, p < 0.01, p < 0.01, p < 0.001, p < 0.001 and p < 0.001 respectively, Fig. 4L, M).

The representative images of JC-1 (membrane potential) are presented in Fig. 4N. The reduced membrane potential values were observed in CSE-exposed cells compared to control cells (p < 0.001, Fig. 4O**)**, which were prevented by treatment with both leflunomide and BGP-15 (both p < 0.001, Fig. 4O**)**.

**Fig. 5:**
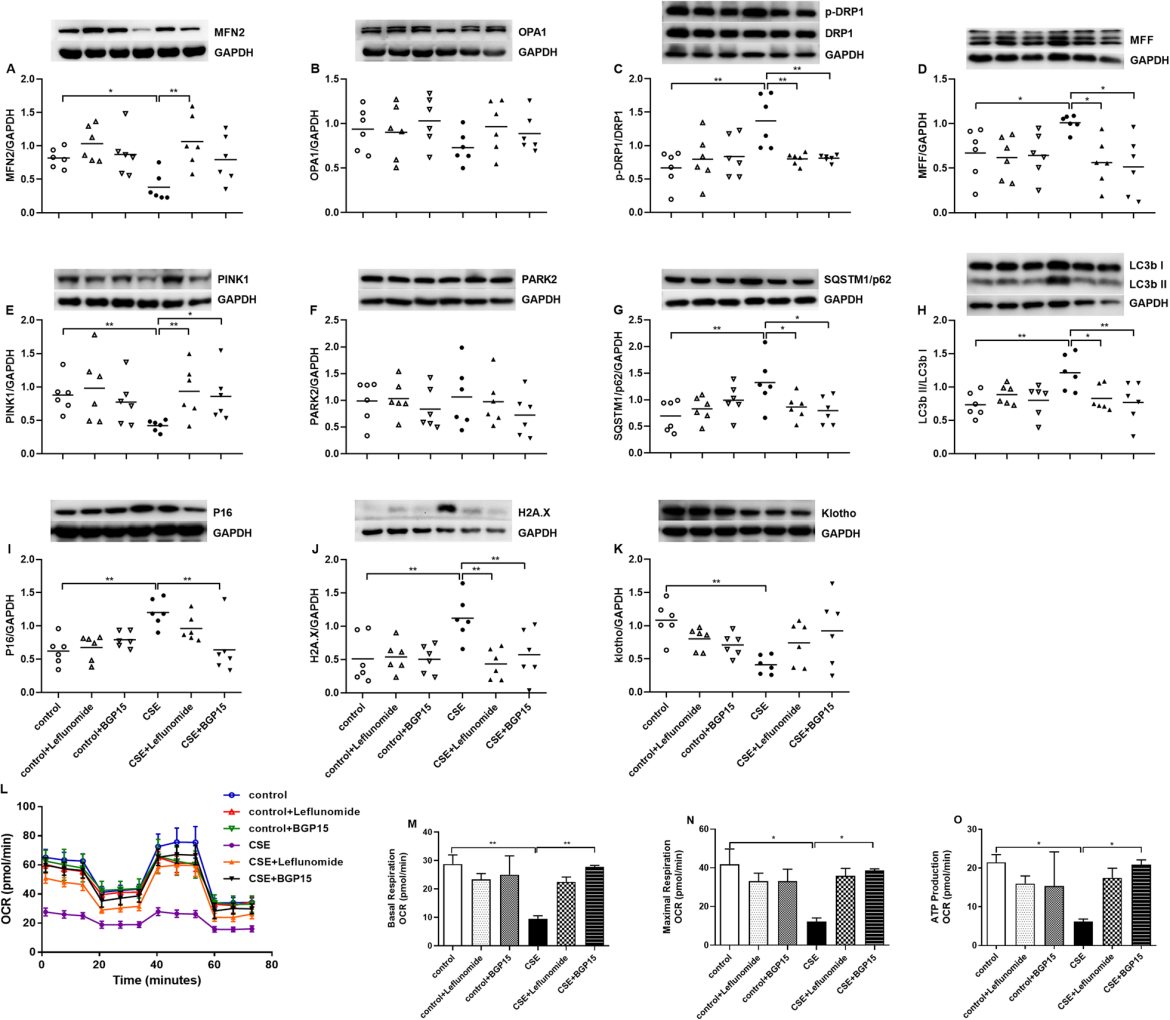
Effects of leflunomide (10µM) and BGP15 (15µM) on mitochondria-related proteins and senescence-related proteins, and oxygen consumption rate (OCR) in A549 cells in the presence and absence cigarette smoke extract (CSE). Mitochondria-related protein levels of MFN2 (**A**) and OPA1 (**B**), phosphorylated-DRP1 (p-DRP1)/DRP1 (**C**), MFF (**D**), PINK1 (**E**), PARK2 (**F**), SQSTM1/p62 (**G**) and LC3b II/ LC3b I (**H**) and senescence-related protein levels of P16 (**I**), H2A.X (**J**) and Klotho (**K**) are measured by Western blot. N = 6 in each group. Every panel incorporates an illustrative Western blot image, and the data are presented as individual and mean values of each protein. Results of mitochondrial activities are presented as oxygen consumption rate (OCR) (**L**), and graphical analysis of basal (**M**) and maximal (**N**) respiration and on ATP production (**O**). N = 4 in each group. ^*^p < 0.05, ^**^p < 0.01, ^***^p < 0.001

The protein expression of MFN2 was reduced in CSE-exposed cells, which was increased by leflunomide treatment (p < 0.05 and p < 0.01, Fig. 5A). No significant difference was observed in OPA1 levels in control cells, leflunomide or BGP15-treated control cells, CSE-exposed cells and leflunomide or BGP15-treated CSE-exposed cells (Fig. 5B). The protein ratios of p-DRP1/DRP1 and the protein levels of MFF were upregulated by CSE exposure (p < 0.01 and p < 0.05 respectively, Fig. 5C, D), and both were inhibited by treatment of both leflunomide and BGP-15 (p < 0.01, p < 0.01, p < 0.05 and p < 0.05 respectively, Fig. 5C, D). Compared to control cells, CSE-exposed cells had reduced protein levels of PINK1, increased protein levels of SQSTM1/p62 and increased ratios of LC3b II/LC3b I (all p < 0.01, Fig. 5E, G, H), while treatment with leflunomide and BGP-15 respectively increased protein levels of PINK1, and reduced protein levels of SQSTM1/p62 and ratios of LC3b II/LC3b I (p < 0.01, p < 0.05, p < 0.05, p < 0.05, p < 0.05 and p < 0.01 respectively, Fig. 5E, G, H). The protein levels of PARK2 remained unchanged among different groups (Fig. 5F).

CSE also promoted lung cellular senescence, as indicated by elevated protein levels of P16 and H2A.X and reduced protein levels of Klotho (all p < 0.01, Fig. 5I–K). BGP15 treatment attenuated P16 protein expression, while treatment with leflunomide and BGP15 respectively attenuated H2A.X protein expression (both p < 0.01 Fig. 5I, J). There is an increasing trend of Klotho protein by treatment with leflunomide and BGP15 (Fig. 5K).

The OCR at baseline (basal) and at maximal respiration together with ATP production were reduced after CSE exposure (p < 0.01, p < 0.05 and p < 0.05 respectively, Fig. 5L–O). Leflunomide exhibited a preventive effect, albeit non-significant, on the CSE-induced inhibition of basal and maximal OCR, as well as ATP production (Fig. 5L–O). BGP15 effectively countered the suppressed basal and maximal OCR and ATP production induced by CSE (p < 0.01, p < 0.05 and p < 0.05 respectively, Fig. 5L–O).

### Effects of overexpression (OE) of MFN2 and OPA1 in CSE-exposed A549 cells

**Fig. 6:**
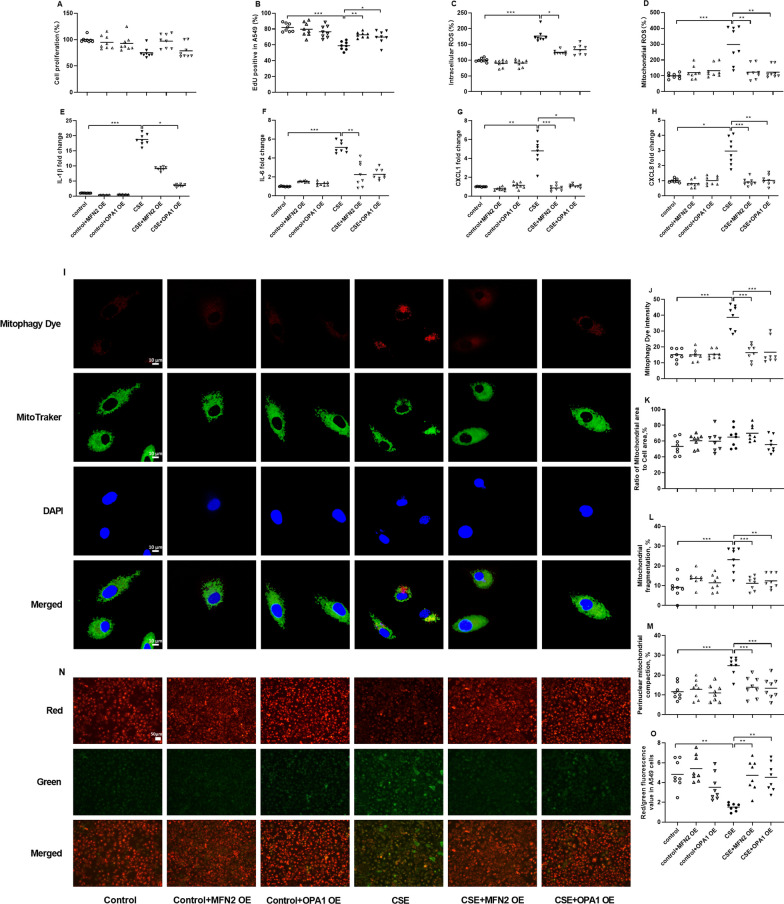
Effects of MFN2 overexpression (OE) and OPA1 OE on cell proliferation, oxidative stress, senescence-associated secretory phenotype (SASP) components mRNA levels and mitochondrial morphology in A549 cells in the presence and absence of cigarette smoke extract (CSE). Cell viability (**A**), cell proliferation (B), intracellular ***(*C**) and mitochondrial ROS (**D**) are respectively detected by CCK-8, Edu staining, DCFH-DA and Mito SOX Red, and data are displayed as percentage changes compared to control cells. Quantitative real-time PCR is employed to measure the mRNA levels of IL-1β (**E**), IL-6 (**F**), CXCL1 (**G**) and CXCL8 (**H**) in relation to β-actin, and data are displayed as individual and mean values of fold changes compared to control cells. N = 6 in each group. Representative confocal microscopy images of the mitophagy dye fluorescence and Mito-Tracker Green staining (**I**, original magnification, ×63). The scale bar is 10 μm. The fluorescent intensity of mitophagy (**J**), the area of mitochondria (**K**), and percentage of mitochondrial fragmentation (**L**) and perinuclear mitochondrial compaction (**M**) are assessed by MitoTracker Green. Representative fluorescent microscopy images of JC-1 staining (**N**) and JC-1 values (**M**). The scale bar is 50 μm. N = 8 in each group. Data are presented as individual and mean values. ^*^p < 0.05, ^**^p < 0.01, ^***^p < 0.001

The CCK-8 assay demonstrated that CSE exposure, MFN2 OE and OPA1 OE had no significant effect on the cell viability (Fig. 6A). However, the EdU staining showed that CSE exposure inhibited the proliferation of A549 cells which could be reversed by both MFN2 OE and OPA1 OE (p < 0.01 and p < 0.05 respectively, Fig. 6B). Intracellular ROS and mtROS levels were increased after CSE exposure (both p < 0.001, Fig. 6C, D). MFN2 OE ameliorated the CSE-enhanced intracellular ROS and mtROS levels, while OPA1 OE attenuated the CSE-enhanced mtROS level (p < 0.05, p < 0.01 and p < 0.01 respectively, Fig. 6C, D).

CSE exposure also led to an elevation in IL-1β, IL-6, CXCL1, and CXCL8 mRNA levels (p < 0.001, p < 0.001, p < 0.01 and p < 0.05 respectively, Fig. 6E-H). OPA1 OE reduced the CSE-enhanced IL-1β mRNA level, while MFN2 OE reduced the CSE-enhanced IL-6 mRNA level (p < 0.05 and p < 0.01, Fig. 6E, F). Both MFN2 OE and OPA1 OE effectively ameliorated the CSE-elevated mRNA levels of CXCL1 and CXCL8 (p < 0.001, p < 0.05, p < 0.001 and p < 0.01 respectively, Fig. 6G, H).

The representative images of mitophagy dye and mitochondrial morphology are shown in Fig. 6I. The intensity of mitophagy fluorescence was increased in CSE-exposed cells, and it was reduced by MFN2 OE or OPA1 OE (all p < 0.001, Fig. 6J). There was no significant difference in mitochondrial area among different groups of A549 cells (Fig. 6K). Mitochondrial fragmentation and perinuclear mitochondrial compaction were increased in CSE-exposed cells, which were inhibited by MFN2 OE or OPA1 OE (p < 0.001, p < 0.001, p < 0.01, p < 0.001, p < 0.001 and p < 0.001 respectively, Fig. 6L, M).

The representative images of JC-1 (membrane potential) are presented in Fig. 6N. The reduced membrane potential values were observed in CSE-exposed cells (p < 0.01, Fig. 6O), which were prevented by treatment with MFN2 OE and OPA1 OE (both p < 0.01, Fig. 6O).

**Fig. 7:**
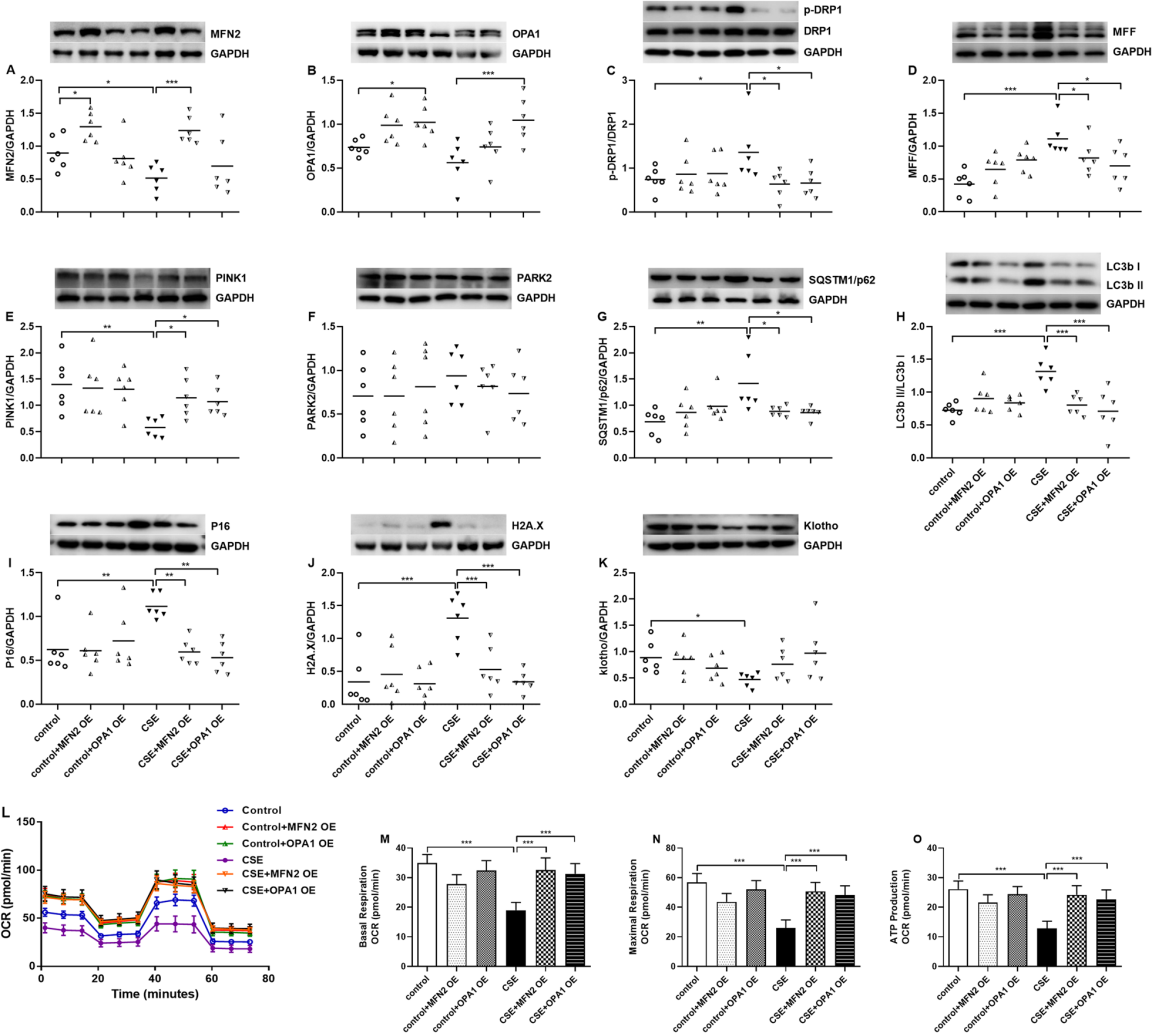
Effects of MFN2 overexpression (OE) and OPA1 OE on mitochondria-related proteins, senescence-related proteins, and oxygen consumption rate (OCR) in A549 cells in the presence and absence of cigarette smoke extract (CSE). Mitochondria-related protein expression levels of MFN2 (**A**) and OPA1 (**B**), phosphorylated-DRP1 (p-DRP1)/DRP1 (**C**), MFF (**D**), PINK1 (**E**), PARK2 (**F**), SQSTM1/p62 (**G**) and LC3b II/ LC3b I (**H**), and senescence-related protein levels of P16 (**I**), H2A.X (**J**) and Klotho (**K**) are measured by Western blot. N = 6 in each group. Every panel incorporates an illustrative Western blot image, and the data are presented as individual and mean values of each protein. Results of mitochondrial activities are presented as oxygen consumption rate (OCR) (**L**) and graphical analysis of basal (**M**) and maximal (**N**) respiration and on ATP production (**O**). N = 4 in each group. ^*^p < 0.05, ^**^p < 0.01, ^***^p < 0.001

The protein expression of MFN2 was decreased in CSE-exposed cells (p < 0.05, Fig. 7A), while no significant change was observed on OPA1 protein expression by CSE (Fig. 7B). The protein levels of MFN2 and OPA1 were increased by MFN2 OE and OPA1 OE in control cells and CSE-exposed cells (p < 0.05, p < 0.001, p < 0.05 and p < 0.001 respectively, Fig. 7A, B).

The protein ratios of p-DRP1/DRP1 and the protein levels of MFF were upregulated by CSE exposure (p < 0.05 and p < 0.001 respectively, Fig. 7C, D), and both were inhibited by MFN2 OE and OPA1 OE treatment (all p < 0.05 respectively, Fig. 7C, D). Compared to control cells, CSE-exposed cells had reduced protein levels of PINK1, increased protein levels of SQSTM1/p62 and increased ratios of LC3b II/LC3b I ( p < 0.01, p < 0.01 and p < 0.001 respectively, Fig. 7E, G, H), while treatment with MFN2 OE and OPA1 OE increased protein levels of PINK1, and reduced protein levels of SQSTM1/p62 and ratios of LC3b II/LC3b I (p < 0.05, p < 0.05, p < 0.05, p < 0.05, p < 0.001 and p < 0.001 respectively, Fig. 7E,G,H). The protein levels of PARK2 remained unchanged among different groups (Fig. 7F).

CSE enhanced protein levels of P16 and H2A.X (p < 0.01 and p < 0.001 respectively, Fig. 7I, J) and reduced that of Klotho (p < 0.05, Fig. 7K). Both MFN2 OE and OPA1 OE attenuated CSE-enhanced protein expression of P16 and H2A.X (p < 0.01, p < 0.01, p < 0.001 and P < 0.001 respectively, Fig. 7I, J). Klotho protein expression was not affected by either MFN2 OE or OPA1 OE (Fig. 7K).

The effect of CSE and OE on OCR is shown in Fig. 7L. In CSE-exposed cells, basal and maximal respiration OCR, as well as ATP production were reduced (all p < 0.001 respectively, Fig. 7M–O). These effects of CSE were prevented in MFN2 OE and OPA1 OE cells (all p < 0.001 respectively, Fig. 7M–O).

## Discussion

Growing evidence supports that lung cellular senescence significantly contributes to the development CS-induced COPD [17]. Mitochondrial dysfunction is recognized as a contributing factor to aging and cellular senescence in COPD pathogenesis. This study unveils a crucial connection between indicators of lung cellular senescence and impaired mitochondrial dynamics and mitophagy in COPD patients. We found increased mitochondrial fragmentation and autophagosomes, impaired mitochondrial function, increased SASP mediators mRNA expression, along with abnormal expression of mitochondria-related proteins (increased levels of DRP1, DRP1 phosphorylation, MFF, PARK2, SQSTM1/p62 and LC3b II /LC3b I, and decreased levels of MFN2, OPA1 and PINK1), elevated senescence-related proteins (P16 and H2A.X) and reduced anti-aging protein (Klotho) in lung tissues of COPD patients. Some similar results were identified in primary ATII cells derived from the lungs of COPD patients. Moreover, our investigation encompassed the utilization of pharmacological induction and genetic overexpression of two essential mitochondrial fusion proteins, MFN2 and OPA1. This strategic intervention resulted in the alleviation of oxidative stress, decrease in SASP mRNA levels, increase in mitochondrial fusion proteins, reduction in mitophagy, decrement in senescence-related proteins and improvement of mitochondrial morphology and OCR in CSE-exposed A549 cells. These results emphasized the potential significance of modulation of MFN2 and OPA1 in preventing mitochondrial dysfunction and lung cellular senescence in COPD, thus preventing the progress of COPD.

CS exposure induces senescence in lung epithelial cells, a phenomenon closely intertwined with oxidative stress and mitochondrial dysfunction and ultimately linked to the development of COPD [18, 19]. The lung aging process in COPD is due to the accumulation of senescent cells in the lung together with the SASP response by the activation of nuclear factor-κB (NF-κB) [20]. This response involves the activation of the NRLP3 inflammasome, which contributes to the release of IL-1β, further exacerbating the SASP response [21]. Although the SASP signaling is designed to prompt the immune system to eliminate senescent cells, the waning efficiency of immune clearance due to aging leads to the accumulation of senescent cells. Consequently, the SASP, operating in a paracrine manner, induces senescence in neighboring cells and intensifies persistent inflammation, thereby fostering a cycle of chronic inflammation [22]. In the current investigation, an elevation in the mRNA expression of SASP components was detected in both CS-induced COPD patients and CSE-exposed A549 cells. There was a decreased expression of Klotho in both lung tissues and primary ATII cells from smokers and COPD patients which is consistent with earlier data [15], and there were enhanced levels of P16 and H2A.X in the lung of COPD patients and of P16 in smokers in comparison to non-smokers. These data confirmed cellular senescence in COPD patients and CSE-induced A549 cells.

Mitochondrial function relies significantly on maintaining a balance between mitochondrial dynamic and mitophagy, both of which exert a significant influence on cellular senescence [23]. Our findings demonstrated that the cellular senescence was correlated with impaired mitochondrial dynamic and enhanced mitophagy. A larger cohort of subjects will allow segregation of the results according to whether COPD and smokers were current or active smokers and address the degree to which the observed changes in senescence markers is due to active smoking rather than COPD itself. A previous study has reported that knockdown of OPA1 or MFNs could increase the production of mitochondrial ROS and percentages of senescent cells in human bronchial epithelial cells (HBECs) [24]. The current investigation also explored the impact of pharmacologic inducer (leflunomide and BGP15) or genetic induction of MFN2 and OPA1 on mitochondrial morphology and function as well as on lung cellular senescence. Both leflunomide and BGP15 treatment not only improved mitochondrial morphology and the oxidative phosphorylation (OXPHOS)-related parameters including intracellular ROS and mtROS, but also attenuated CSE-induced mRNA levels of SASP components and protein expression of P16 and H2A.X in A549 cells, while BGP15 improved basal and maximal OCR and ATP production. These OCR changes were more obviously in those reported after lentiviral-mediated overexpression of MFN2 and OPA1.

Our observation concerning the increased levels of p-DRP1/DRP1, DRP1 and MFF and the reduced levels of MFN2 and OPA1 in lung tissues and ATII cells of COPD patients and after CSE smoke exposure of A549 cells is in concordance with several other studies. Elevated mRNA and protein levels of DRP1 and FIS1, along with reduced expression of MFN2 and OPA1, were observed in human airway smooth muscle cells following a 24-hour exposure to CSE [25]. Similarly, augmented DRP1 protein levels and diminished MFN2 protein levels were measured in 15-day CSE exposure of primary lung epithelial cells [26]. Reduced expression of MFN1, MFN2 and OPA1 were also seen in alveolar epithelial cells in emphysema/COPD patients [27]. Other conflicting data have been reported mainly in models of CSE exposure [17, 19], but this may be explained by different CSE concentrations and exposure duration. In addition, in our CSE exposure cell models, the impaired mitochondrial fission and fusion protein levels could be prevented by up-regulation of MFN2 and OPA1.

In this study, the enhanced mitophagy was evidenced by the formation of autophagosomes and the elevated intensity of mitophagy fluorescence, along with the aberrant expression of mitophagy-related protein. Typically, PINK1 facilitates the recruitment of PARK2 to mitochondria, initiating the mitophagy process, wherein PARK2 can emerge as the decisive factor, exerting a more pronounced influence than PINK1 in COPD pathogenesis [17]. Subsequently, PARK2 ubiquitinates and degrades MFN1/2, and interacts with LC3b II through the intermediary SQSTM1/p62 adaptor protein. This culminates in the formation of autophagosomes and triggers mitophagy, a process interlinked with oxidative stress [28]. Impaired PARK2 translocation to damaged mitochondria was noted in the lung tissues of emphysema-afflicted mice, chronic smokers and COPD patients [29]. Both decreased PARK2 protein [30] and impaired autophagy [31, 32] were observed in the lungs of COPD patients. In CS-exposed mice, there was a cooperative rise in the expression of PINK1 and PARK2 within the lung tissues [33]. Acute exposure of whole CS or CSE to primary HBECs induced the autophagy-related proteins such as SQSTM1/p62 and LC3b [34]. In the present study, there were decreased PINK1 protein levels, and increased PARK2, SQSTM1/p62 protein levels and increased ratios of LC3b II/I. Up-regulation of MFN2 and OPA1 effectively prevented the CSE-induced mitophagy in A549 cells. Nevertheless, the activity of mitophagy in COPD remains controversial, which may be due to difference in selection of patients or modelling methods, and requires further investigation.

Several in vivo and in vitro investigations have demonstrated mitochondrial dysfunction, including changes in mitochondrial morphology, impaired OXPHOS and energy production, declined mitochondrial membrane potential and increased mtROS production, as a pathological factor in the progression of COPD [35]. Morphologically, mitochondria in bronchiolar epithelial cells were prone to be more fragmented and shorter in average size in COPD than that of control cases [24]. The present study confirmed this data with increased numbers of fragmented mitochondria per cell and decreased mitochondrial size in lung tissues of COPD patients, and with increased mitochondrial fragmentation and declined mitochondrial membrane potential in primary ATII cells of COPD patients as well as in CSE-exposed cells, which may also indicate ferroptosis in COPD [36]. These morphological changes in mitochondria in COPD patients could be related to the increased expression of DRP1 and MFF and decreased expression of OPA1 and MFN2 in the lung of COPD patients.

OXPHOS in mammals is regulated by the electron transport chain (ETC) formed by complexes I-V and two mobile electron carriers [37]. Complex I and III are two major sources in the generation of ROS, while Complex V finally utilizes the proton gradient to convert ADP to ATP. There were reduced complex I, III and V activities in the lung of smokers and COPD patients as compared to non-smokers. Additionally, we identified reduced OCR and ATP production in CSE-exposed A549 cells in our study, which indicate impaired OXPHOS and energy production. These changes of mitochondria in COPD patients could be related to the abnormal expression of mitochondrial dynamics in lung tissues as well as in primary ATII cells of COPD patients.

Although this study has several strengths, we note some limitations. First, the subject cohorts are relatively small, which precludes separating subjects into current smokers and ex-smokers. Second, all subjects were males in the study because most of patients with both COPD and suspected lung cancer are male smokers, therefore, Subsequent investigations will be required to ascertain whether comparable outcomes manifest in females and in an independent validation group. Third, we repeated some results in primary human ATII cells. It would also be interesting to determine whether similar responses occur in primary human bronchial epithelial cells or lung fibroblasts. Last, we need to perform such interventions in COPD mouse models, which is under planning.

In conclusion, we explored the link between mitochondrial dynamics and lung cellular senescence in the pathogenesis of COPD. Deficiency of MFN2 and OPA1 induced by CS exposure leads to mitochondrial dysfunction and lung cellular senescence in COPD. Up-regulation of MFN2 and OPA1 could be a novel and promising therapeutic approach for delaying or reversing COPD progress.

### Supplementary Information

**Additional file 1**. Additional file materials and methods. A detailed materials and methods

## Data Availability

The datasets used or analyzed during the current study are available from the corresponding author on reasonable request.

## Abbreviations

COPD: Chronic obstructive pulmonary disease
MFN: Mitofusin
OPA1: Optic atrophy 1
CS: Cigarette smoke
CSE: CS extract
DRP1: Dynamin-related protein 1
FIS1: Fission 1
MFF: Mitochondria fission factor
MID49: Mitochondrial dynamics protein of 49 kDa
PINK1: PTEN-induced kinase 1
PARK2: Parkinson disease protein 2
SQSTM1/p62: Sequestosome-1
LC3: Microtubule-associated protein 1 A/1B-light chain 3
ATII: Alveolar type II
ATM: Ataxia-telangiectasia mutated
H2A.X: H2A histone family member X
Rb: Retinoblastoma
SASP: Senescence-associated secretory phenotype
IL: Interleukin
CXCL: (C-X-C motif) ligand
OE: Overexpression
CAT: COPD assessment test
mMRC: modified Medical Research Council
FEV1: Forced expiratory volume 1s
FVC: Forced vital capacity
TEM: Transmission electron microscopy
mtROS: mitochondrial ROS
MRC: Mitochondrial respiratory chain
OCR: Oxygen consumption rate
BMI: Body mass index
HBECs: Human bronchial epithelial cells
OXPHOS: Oxidative phosphorylation
