# Supplementary material for: Role of mitochondrial fusion proteins MFN2 and OPA1 on lung cellular senescence in chronic obstructive pulmonary disease
Source: Respir Res. 2023 Dec 18;24:319. doi: 10.1186/s12931-023-02634-9 (PMC10726594; doi:10.1186/s12931-023-02634-9)
Supplement: Supplementary file 1 — Additional file 1. Additional file materials and methods. A detailed materials and methods [file 12931_2023_2634_MOESM1_ESM.docx]

**Additional file Materials and Methods**

**Collection of human lung tissues**

This research was approved by the Institutional Ethics Committee (No. KS1969) and written informed consent was signed by each subject. Lung tissues were obtained from newly diagnosed COPD patients or control subjects of no history of obstructive airways diseases with lung nodules or suspected lung cancer undergoing lung resection between July and August 2020 in Shanghai Chest hospital (16). Visually normal tissue samples were dissected as far as possible from the nodular or cancerous tissue, and were immediately transferred in liquid nitrogen and stored at −80°C until being analyzed. A total of 40 subjects were divided into non-smokers (n=10), smokers without obstruction (n=10) and COPD (n=20) patients through a questionnaire and lung function tests. As the COPD patients and smokers were mostly male, we made all subjects including controls men in our research to avoid the influence by gender. Both smokers and COPD patients were active smokers. In this questionnaire, clinical information including sex, age, smoking index, treatment status, lung function, COPD Assessment Test (CAT) scoring and the modified Medical Research Council (mMRC) dyspnea score was obtained. The diagnosis of COPD was based on the criteria of the Global Initiative for Chronic Obstructive Lung Disease (GOLD), namely a post-bronchodilator ratio of forced expiratory volume 1s (FEV1) to forced vital capacity (FVC) <70% using a body plethysmograph (MasterScreen Body/Diff, Jaeger, Hoechberg, Germany).

**Transmission electron microscopy (TEM) analysis in ATII cells of lung tissues**

The pieces of lung were fixed in 2.5% glutaraldehyde for 4 h before incubation in 1% osmium tetroxide for 2h at 4°C. Dehydration was accomplished with increasing concentrations of ethanol (30-100%) and then replaced with propylene oxide for 10 min. The dehydrated tissues were immersed in a mixture of propylene oxide and SPI-pon812 embedding agent (SPI supplies, West Chester, PA, USA) overnight at room temperature for soaking and embedding. After high temperature polymerization, ultrathin sections (70-80nm) were obtained by using a diamond knife (Nidau, Switzerland) and Leica EM UC7 ultramicrotome (Wetzlar, Germany). Finally, the ultrathin sections were stained with uranyl acetate and lead citrate, and then observed by TEM (JEOL-1400 flash, Akishima, Tokyo, Japan). The morphology was qualified by Image J analysis (National Institute of Health, Bethesda, USA). Freehand tool was used to trace the outer mitochondrial membrane of each mitochondrion to measure area, circularity and perimeter while a straight line along the major axis of each mitochondrion was drawn to measure length.

**Isolation and culture of primary ATII cell**

Under sterile conditions, the specimen of distal portions of normal lung tissue was obtained from patients undergoing lung resection (Shanghai Chest Hospital). The specimen was chopped into 1 cm^3^ in size and washed with Hank's Balanced Salt Solution (HBSS, Corning) until clear. The lung pieces were minced (0.5 mm^3^) with scissors and incubated in a solution containing 4 mL of 0.25% trypsin (Gibco) and 4ml of 0.1% collagenase type I (Life technologies) for 30 min at 37°C, and gently pipetted with Pasteur pipet (Thermo Fisher) for 2 minutes every 5 minutes. The enzymatic activity was stopped using 8 mL DMEM/F-12K (Gibco; 10% FBS, Gibco) and 4 mL DNase I (10,000 U/mL, KeyGen biotechnology), and the suspension was pipetted for 10 minutes. The suspension was filtered through cell strainers at the size of 150μm (Pluriselect) and 75μm (WHB biotechnology) in tandem to collect the crude cell suspension.

After filtration, the residual lung tissue was digested and filtered again through the above steps. The cell suspensions obtained from the two filtrations were centrifuged at 2500 r/min for 5 min. The cells were resuspended with DMEM/F12 complete medium, and transferred to a culture dish and incubated at 37°C, 5% CO_2_ for 1-2 h. The unadhered cells were aspirated and centrifuged at 1 000 r/min for 5 min. This differential adhesion procedure was repeated three times, and then the AEII cells were gently collected and centrifuged at 1 000 r/min for 5 min.

8-10 ml of cell suspension was added to the culture dish coated with mouse IgG (Sigma), which was dissolved in 50 mM Tris buffer (pH 9.5, Beyotime) at 0.5-1.0mg/ml, at 37℃, 5% CO_2_ for 3h, the unadhered cells were aspirated and then centrifuged at 400×g for 8 min. Cells were resuspended in DMEM/F12K medium with 20% fetal bovine serum (FBS), 200U/ml penicillin and 200μg/ml streptomycin, and 2.0~3.0×10^6^/ml cells were incubated in 35mm culture dish. The medium was changed every other day. The cells were cultured until they were in good condition, and subsequent experiments were carried out.

**Cell line culture, CSE preparation and exposure, pharmacologic and genetic induction**

A549 cells (Shanghai Institutes for Biological Sciences, China Academy of Science, Shanghai) were cultured in F12K medium (Procell Life Science & Technology, Wuhan, China) with 10% FBS, 100U/ml penicillin and 100μg/ml streptomycin at 37°C in 5% CO_2_. CSE was freshly prepared on the day of each experiment as previously described (8). Two burning cigarettes (Marlboro Red Label, Longyan Tobacco Industrial, Fujian, China) without filters were sucked at a constant flow rate into a syringe and then bubbled into a tube containing 10mL serum-free medium. The CSE solution was sterilized using a 0.22μm filter and the pH was adjusted to 7.4. This CSE solution was considered 100% CSE and was further diluted with serum-free media to the desired concentrations and used to treat cells.

Following observations from a preliminary study, A549 cells were treated with 10% CSE to induce cell injury. Cells were pretreated with 10μM of leflunomide (MFN2 promoter) (#S1247，Selleck, Shanghai, China) or 15μM BGP15 (OPA1 promoter) (#S8370，Selleck, Shanghai, China) for 2h and then were exposed to vehicle or CSE for another 24h. Human MFN2-OE and OPA1-OE plasmid sequences were commercially designed (Lncbio-technology, Xuhui, Shanghai, China). Lentivirus packaging was performed in 293T cells using Zorin virus packaging kit (Shanghai Zorin Biological Technology, Shanghai, China). After the lentivirus was prepared, the cells were stably transfected using Polybrene, screened using antibiotic puromycin dihydrochloride (Shanghai Zorin Biological Technology) resistance to obtain MFN2- and OPA1- overexpressing cells.

**Mitochondrial potential, mitophagy activity and morphology analysis**

Primary ATII cells or A549 cells were seeded in 6-well plates with a density of 2×10^5^ cells/well. Cells were stained with 10 mg/ml JC-1 ( Thermo Fisher Scientiﬁc, MA, USA ) at 37 °C in the dark for 10 min to label the mitochondria. Normal mitochondrial potential showed red ﬂuorescence while damaged mitochondrial potential showed green ﬂuorescence. The red/green ﬂuorescence value was used to quantify the mitochondrial membrane potential.

Cells were seeded in confocal dishes with a density of 5×10^4^cells/ml. A mitophagy detection kit (Dojindo, Kumamoto, Japan) was performed for mitophagy activity following the instructions from the manufacturer. Briefly, A549 cells were stained with 100 nmol/l Mitophagy Dye working solution at 37°C for 30 minutes, and then incubated with 10 μmol/l carbonyl cyanide 3-chlorophenylhydrazone (CCCP) (Solarbio Life Sciences, Beijing, China) for 24h, A549 cells were imaged by a confocal laser scanning microscope(Leica, Wetzlar, Germany). Cells were then subsequently incubated with 200nM MitoTracker Green staining and 1μg/ml DAPI for measurement of morphology. The ratio of MitoTracker area to cell area, mitochondrial fragmentation percentage and perinuclear mitochondrial compaction percentage were calculated as previously described.Mitochondrial fragmentation was judged if >90% of the mitochondria in the cytoplasm outside of the perinuclear compaction were punctate or circular, and mitochondrial perinuclear compaction was judged if >90% of the mitochondria accumulated in the perinuclear area.

**Cell viability and cell proliferation assay**

The effect of vehicle or CSE on cell viability was measured by cell counting kit-8 (CCK8, Dojindo, Kumamoto, Japan). Cells were cultured in a 96-well plate with a density of 5×10^4^cells/ml overnight at 37°C before exposure to vehicle or CSE. After 24h incubation, cells were incubated with CCK8 working solution for 2h at 37°C following the Manufacturer's protocol. The absorbance at 450 nm was measured by microplate reader iMark (Molecular Devices, Sunnyvale, CA, USA).

The effect of vehicle or CSE on cell proliferation was confirmed by 5-Ethynyl-2’-deoxyuridine (EdU) incorporation assay, with the concentration of 10 μM EdU. Cells were cultured in 24-well plates, and then incubated with EdU Cell Proliferation Kit with DAB (Beyotime, Shanghai, China) according to the manufacturer’s instructions.

**Measurement of intracellular ROS and** **mitochondrial ROS (mtROS) in cells**

Intracellular ROS generation was detected using DCFH-DA (Sigma-Aldrich, St. Louis, MO, USA). Briefly, the cells were seeded in 96-well black plates with a density of 5×10^4^ /ml with 6 parallel wells in each group. Cells were stained with 10μM DCFH-DA at 37°C in the dark for 15 min. Then cells were washed with serum-free F12K for three times, the level of ROS was determined using a fluorescence plate reader (Molecular Devices) at 488/525 nm. mtROS level was measured using Mito SOX Red (Invitrogen, Life Technologies, Carlsbad, CA, USA). Briefly, the cells were seeded in 96-well black plates with a density of 5×10^4^ /ml with 6 parallel wells in each group. Cells were incubated with 5mmol/L Mito SOX Red probe for 10 min at 37℃. The cells were washed twice with PBS, and red fluorescence was determined at 510/580 nm using a fluorescence plate reader.

**Quantitative real-time PCR in lung tissues and cells**

Total RNA was extracted from human lung tissues and A549 cells using TRIzol (Vazyme, Nanjing, Jiangsu, China) and then its concentration and purity were assessed. 1 μg total RNA was reverse transcribed into cDNA and quantitative real-time PCR was performed with ChamQ Universal SYBR qPCR Master Mix (Vazyme) in an ABI ViiATM 7 System (Applied Biosystems, Foster City, CA, USA). The reaction conditions included 95°C for 30s, followed by 40 cycles of 95°C for 10s and 60°C for 30s, with a final cycle of 95°C for 15s, 60°C for 60s and 95°C for 15s. The primer sequences of the cytokines and β-actin are shown in **Table 1**.

**Table S1.** Primer sequences of cytokines and β-actin

| IL-1β | Forward | 5’- TCGCAGCAGCACATCAACAAGAG -3’ |
| --- | --- | --- |
|  | Reverse | 5’- AGGTCCACGGGAAAGACACAGG -3’ |
| IL-6 | Forward | 5’- CACTGGTCTTTTGGAGTTTGAG -3’ |
|  | Reverse | 5’- GGACTTTTGTACTCATCTGCAC -3’ |
| CXCL1 | Forward | 5’- AAGAACATCCAAAGTGTGAACG -3 |
|  | Reverse | 5’- CACTGTTCAGCATCTTTTCGAT -3’ |
| CXCL8 | Forward | 5’- AACTGAGAGTGATTGAGAGTGG -3’ |
|  | Reverse | 5’- ATGAATTCTCAGCCCTCTTCAA -3’ |
| β-actin | Forward | 5’- GGCCAACCGCGAGAAGATGAC -3' |
|  | Reverse | 5’- GGATAGCACAGCCTGGATAGCAAC -3' |

**Western Blot analysis in lung tissues and cells**

Lung tissues, primary ATII cell**s** and A549 cells were homogenized and lysed in RIPA Lysis Buffer (Beyotime) for 30 min on ice to obtain total proteins, which were measured by a BCA kit (Beyotime). 30μg protein from each sample was separated by 12% SDS page at 60 V for 30 min and 120V for 60 min before being blotted onto a 0.45μm PVDF membrane (Millipore, Billerica, MA, USA). Membranes were incubated with primary antibodies against DRP1 (#8570, 1:1000, Cell Signaling Technology, Danvers, MA, USA), phosphorylated-DRP1 (p-DRP1) (Ser616) (#3455, 1:1000, Cell Signaling Technology, Danvers, MA, USA) MFF (#84580, 1:1000, Cell Signaling Technology), OPA1 (#80471, 1:1000, Cell Signaling Technology), MFN2 (#9482, 1:1000, Cell Signaling Technology), PINK1 (#ab23707, 1:1000, Abcam, Cambridge, MA, USA), PARK2 (#ab77924, 1:1000, Abcam), SQSTM1/p62 (#ab56416, 1:1000, Abcam), LC3b (#ab192890, 1:1000, Abcam), P16 (#ab51243, 1:1000, Abcam), H2AX (#ab2893, 1:1000, Abcam), Klotho (#500-P296, 1:1000, Proteintech, Wuhan, Hubei, China) and GAPDH (#10494-1-AP, 1:1000, Proteintech) overnight at 4°C, and then incubated with the secondary antibodies, HRP-conjugated goat anti-rabbit IgG (#7074, 1:2000, Cell Signaling Technology, Danvers, MA, USA) or HRP-conjugated goat anti-mouse IgG (#7076, 1:2000, Cell Signaling Technology, Danvers, MA, USA) for 2 h at room temperature. Bands were developed by ECL chemiluminescent substrate (Millipore, Billerica, MA, USA).

**Assay of** **mitochondrial respiratory chain (MRC) activities in lung tissues**

The activities of MRC complexes I, III and V were assessed using an activity assay kit (#BC0515, #BC3245 and #BC1445, Solarbio Life Sciences, Beijing, China) according to the manufacturer’s instructions. Lung tissues were homogenized in isolation buffer to obtain mitochondria by centrifuging at different speeds following the instruction from the manufacturer. The activity of MRC complex I was determined at 340nm, MRC complex III was determined at 550nm and MRC complex V was determined at 660nm using a spectrophotomer (Thermo Fisher Scientific, MA, USA). The protein concentrations of the lung tissue homogenates were measured by a BCA kit (Beyotime). The consumption of 1 nmol NADH (Nicotinamide adenine dinucleotide, NADH) per minute per mg protein was calculated as the activity of MRC complex I. The generation of reduced cytochrome c per minute per mg protein was calculated as the activity of MRC complex III. The generation of Pi per minute per mg protein was calculated as the activity of MRC complex III. The MRC complexes I, III and V activities in the lungs of non-smokers were all set to 100%.

**Measurement of mitochondrial** **oxygen consumption rate (OCR) in cells**

A549 cells were seeded on Seahorse XFe96 plates at a density of 10000 cells/well. The OCR was measured using an XFe96 Extracellular Flux Analyzer (Seahorse Bioscience, North Billerica, MA, USA) according to the Manufacturer’s instructions. The following inhibitors were added sequentially: 1.5μM oligomycin (an ATP uncoupler) (Seahorse Bioscience), 1μM carbonyl cyanide p-trifluoromethoxyphenylhydrazone (an ETC accelerator) (Seahorse Bioscience), 0.5μM antimycin A (a complex III inhibitor) (Seahorse Bioscience), and 0.5μM rotenone (a complex I inhibitor) (Seahorse Bioscience). OCR was calculated using the standard XFe96 Extracellular Flux Analyzer protocol.

**Statistical analysis.**

Data are presented as mean ± SD. SPSS software 20.0 (IBM, NY, USA) was used for correlation analysis by Pearson’s test for normally distributed data or the Spearman’s rank test for non-normally distributed data, and for multiple composition ratio comparison using Fisher’s exact test. GraphPad Prism 8 was used to compare multiple groups by One-way ANOVA with Bonferroni’s post hoc test (for equal variance) or Dunnett’s T3 post hoc test (for unequal variance) followed by a correction for multiple comparisons by controlling the false discovery rate with the two-stage step-up method of Benjamini, Krieger, and Yekutieli. P<0.05 was considered significant.
